# Mapping randomized controlled trials of treatments for eczema - The GREAT database (The Global Resource of Eczema Trials: a collection of key data on randomized controlled trials of treatments for eczema from 2000 to 2010)

**DOI:** 10.1186/1471-5945-11-10

**Published:** 2011-05-18

**Authors:** Helen Nankervis, Alan Maplethorpe, Hywel C Williams

**Affiliations:** 1 Centre of Evidence Based Dermatology, Room A103, King's Meadow Campus, University of Nottingham, Lenton Lane, Nottingham, NG7 2NR, UK; 2 Clinical Trials Unit, University of Nottingham, Queens Medical Centre Campus, Medical School, Derby Road, Nottingham, NG7 2UH, UK

## Abstract

**Background:**

Massive duplication of effort occurs when researchers all over the world undertake extensive searches for randomized controlled trials when preparing systematic reviews, when developing evidence-based guidelines and when applying for research funding for eczema treatments. Such duplication wastes valuable resources.

Searching for randomized controlled trials of eczema is a laborious task involving scrutiny of thousands of individual references from diverse electronic databases in order to obtain a few papers of interest. Clinicians and patients who wish to find out more about a particular treatment are at risk of missing the relevant evidence if they are not trained in electronic bibliographic searching. Systematic reviews cannot be relied upon to comprehensively inform current optimal eczema treatments due to incomplete coverage and because many may be out of date.

An international, publically available and comprehensive resource which brings together all randomized controlled trials on eczema treatment using a highly sensitive search has the potential to release more filtered knowledge about patient care to those who need it most and to significantly shorten the duration and costs of many clinical eczema research and guideline projects.

**Description:**

The Global Resource of EczemA Trials brings together information on all randomized controlled trials of eczema treatments published from the beginning of 2000 up to the end of 2010 and will be updated every month.

We searched the Cochrane Central Register of Controlled Trials in *The Cochrane Library *and the Cochrane Skin Group Specialised Register, MEDLINE, EMBASE, LILACS, AMED and CINHAL databases. We included 268 RCTs (24^th ^March 2011) covering over 70 different treatment interventions.

The structure of the Global Resource of Eczema Trials allows the user as much, or as little, specificity when retrieving information on trials as they wish, in an easy to use format. For each trial, the database gives the citation for the published report and also provides enough information to enable a user to decide whether the trial is worth further scrutiny.

**Conclusions:**

The Global Resource of Eczema Trials has been created to facilitate knowledge mobilization into healthcare and to reduce wastage of research time through unnecessary duplication. The collective time saved by research groups around the world can now be used to make strides in optimising the treatment of eczema, in order to further benefit people with eczema. The database can be accessed free of charge at http://www.greatdatabase.org.uk

## Background

Eczema [1] is a complex, chronic and relapsing inflammatory skin disease affecting around 2 to 20% of children worldwide [2,3]. The prevalence of eczema is increasing [4], especially in children, although the reasons for this increase are unclear. The constant itching, sleep loss and visible stigmata of eczema can seriously affect the quality of life of a child and family [5]. The disease also can lead to high socioeconomic costs [6]. Eczema, as defined by the World Allergy Organisation, encompasses both atopic and non-atopic eczema and is commonly referred to as atopic eczema or atopic dermatitis. Contact dermatitis and other forms of dermatitis are considered to be separate conditions and are not covered by the scope of this research [1].

### Finding a needle in a haystack - searching and filtering

Eczema research is conducted all over the world: A prerequisite for much of this research such as applying for funding, systematic reviews and developing clinical guidelines, is to search for randomized controlled trials (RCTs).

There are at least 48 (5 Cochrane and 43 non-Cochrane) systematic reviews on treatments for eczema covering a large number of eczema treatment areas which have been mapped in our department on behalf of NHS Evidence-skin disorders [7]. Whilst the coverage of topic areas is encouraging, many interventions such as intravenous immunoglobulin, liquorice extract, and ketoconazole are currently not included in any form of systematic review, and many existing systematic reviews are out of date [8].

Searching is a laborious task involving wading through hundreds and sometimes thousands of individual references online followed by scrutiny of full paper copies of studies and their associated citation lists in order to obtain a few papers of interest [9]. Often the use of a very sensitive search strategy is necessary in order to be as inclusive as possible, as is the case when conducting a Cochrane systematic review [10]. The more sensitive the search, the more complete the research, however, this adds to the number of references which need to be manually filtered, thereby increasing the time taken to complete a project.

A resource which brings together all RCTs on eczema treatment using a highly sensitive, comprehensive search has the potential to significantly shorten the length of many eczema research projects. Just as some countries like the UK host national reference collections of entities such as plant species in the National Collection of Dahlias [11], our idea is to host a similar reference collection of eczema RCTs that is freely accessible, comprehensive, easily searched and complete that can be used for international eczema research, in the hope that it will lead to improvement in patient care.

The Global Resource of EczemA Trials (GREAT) database brings together information on all RCTs of eczema treatments published from the beginning of 2000 for the first time. The database currently holds records of all RCTs published and indexed up to the end of 2010 and will be updated on an ongoing basis. The database is restricted to RCTs from 2000 onwards as information from trials published before this year can be found in the Health Technology Assessment (HTA) systematic review on treatments for atopic eczema [12], a highly accessed HTA monograph at the time.

This systematic review included all randomized controlled trials of eczema treatments involving people with established eczema. The extracted information from the trials was presented in concise sections summarising the benefits and harms for each treatment, with comments on the quality of reporting of the trials. Information was also summarised in tables for many of the treatments reviewed.

The search strategy for Global Resource of Eczema Trials is very similar to the searches conducted for this HTA review.

## Construction and Content

To be included in the GREAT database, a trial has to fulfil the following broad criteria:

1. The trial report must include a clear mention of randomisation

2. The participants must have established eczema either diagnosed by a clinician or using the Hanifin and Rajka criteria [13] or the UK Working party criteria [14], or criteria very close to these.

3. There must be prospective allocation of people with eczema to two or more interventions

4. The trial must measure at least one efficacy outcome

### Search strategy

To ensure that as many RCTs as possible were identified, we searched the Cochrane Central Register of Controlled Trials (CENTRAL) in *The Cochrane Library *and the Cochrane Skin Group Specialised Register, which includes handsearching of dermatology conference proceedings, as well as MEDLINE (Ovid), EMBASE (Ovid) (Table 1), LILACS, AMED and CINAHL databases from the beginning of 2000 to the end of 2010.

**Table 1:** EMBASE search strategy

1. random$.mp.
2. factorial$.mp.

3. (crossover$ or cross-over$).mp.

4. placebo$.mp. or PLACEBO/

5. (doubl$ adj blind$).mp.

6. (singl$ adj blind$).mp.

7. (assign$ or allocat$).mp.

8. volunteer$.mp. or VOLUNTEER/

9. Crossover Procedure/

10. Double Blind Procedure/

11. Randomized Controlled Trial/

12. Single Blind Procedure/

13. 1 or 2 or 3 or 4 or 5 or 6 or 7 or 8 or 9 or 10 or 11 or 12

14. exp Dermatitis, Atopic/

15. atopic dermatitis.mp.

16. atopic eczema.mp.

17. exp NEURODERMATITIS/

18. neurodermatitis.mp.

19. infantile eczema.mp.

20. childhood eczema.mp.

21. (besnier$ and prurigo).mp.

22. eczema.mp. or exp Eczema/

23. 21 or 17 or 20 or 15 or 14 or 22 or 18 or 16 or 19

24. 23 and 13

The searches for MEDLINE and EMBASE were based on the Cochrane highly sensitive search strategy for RCTs [10], combined with a list of known terms for eczema, intended to be as comprehensive as possible.

## Utility and Discussion

The GREAT database has been constructed with ease of use as its primary objective. The structure of the database allows the user as much, or as little, specificity as they wish. The GREAT database website scores 94% for accessibility using the LIDA instrument, a tool which assesses accessibility by looking at page setup, access restrictions, amount of outdated code and compatibility with NHS directives [15]. The website is already being accessed in the UK as well as around the world in countries such as Sudan, Germany, Ireland, United States, Poland, France and Spain [16].

**Figure 1:**
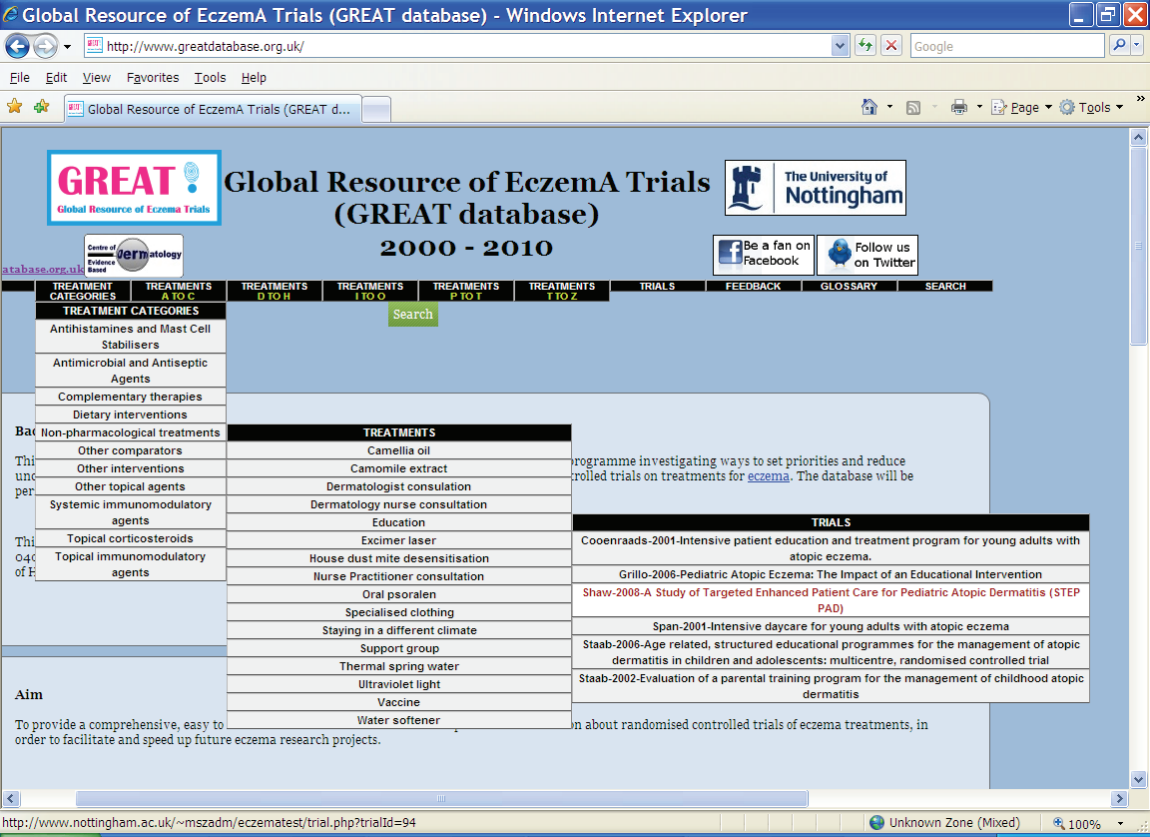
**The GREAT database hierarchy is available on drop down menus**.

If a particular treatment area (such as non-pharmacological treatments) is of interest, the menus across the top of every page offer a list of treatment categories from which it is possible to see a list of all treatments in that category. From the list of treatments, a specific treatment of interest can be selected, which will give a list of the trials looking at that treatment. Selecting a trial will give access to the trial information (Figure 1).

**Figure 2:**
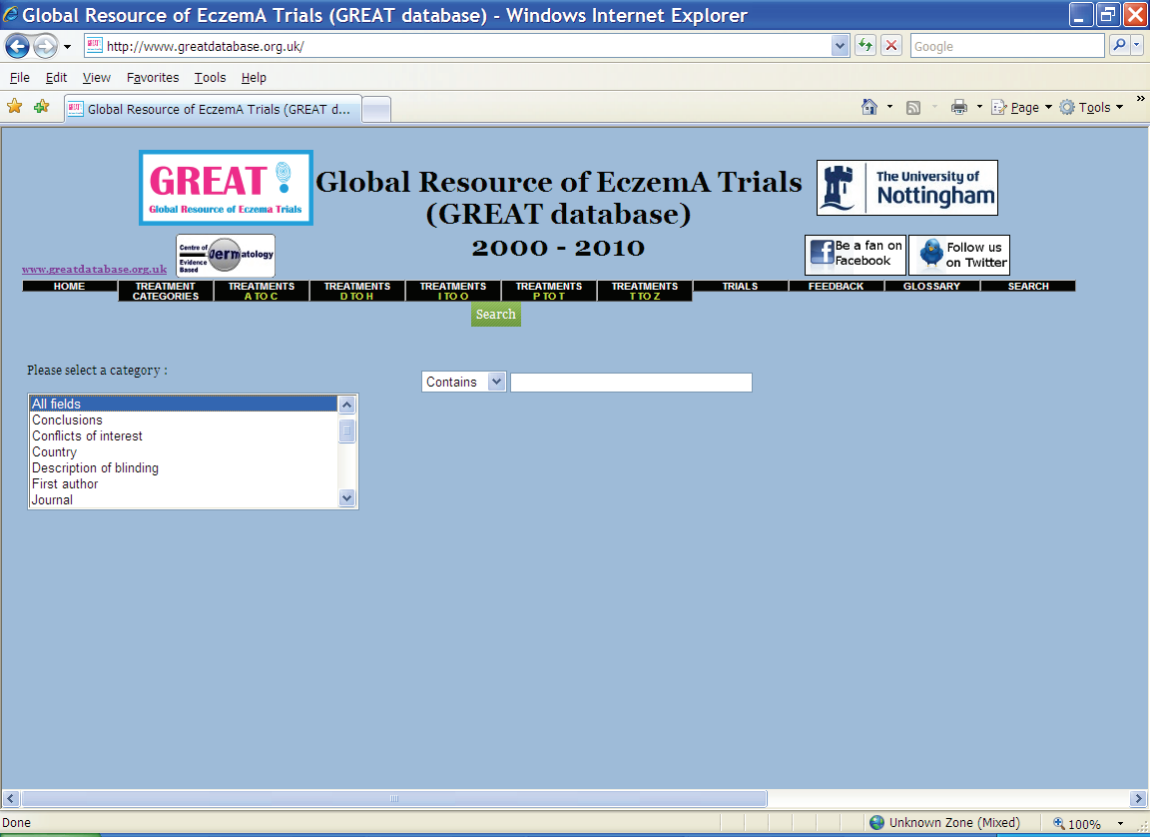
**The GREAT database search facility**.

If a particular trial is of interest and at least one piece of information is known, such as the author or title, the 'trials' page or the 'search' page allows the trial details to be accessed directly. The search facility also allows the user to search for a term of interest, such as 'SCORAD' or 'double blind' and quickly have access to a list of trials whose records contain the term (Figure 2).

All levels of specificity are catered for: a search for all trials from a certain year is possible through buttons on the trials page, as are an alphabetical journal search and first author search from this page.

### Trial information

**Figure 3:**
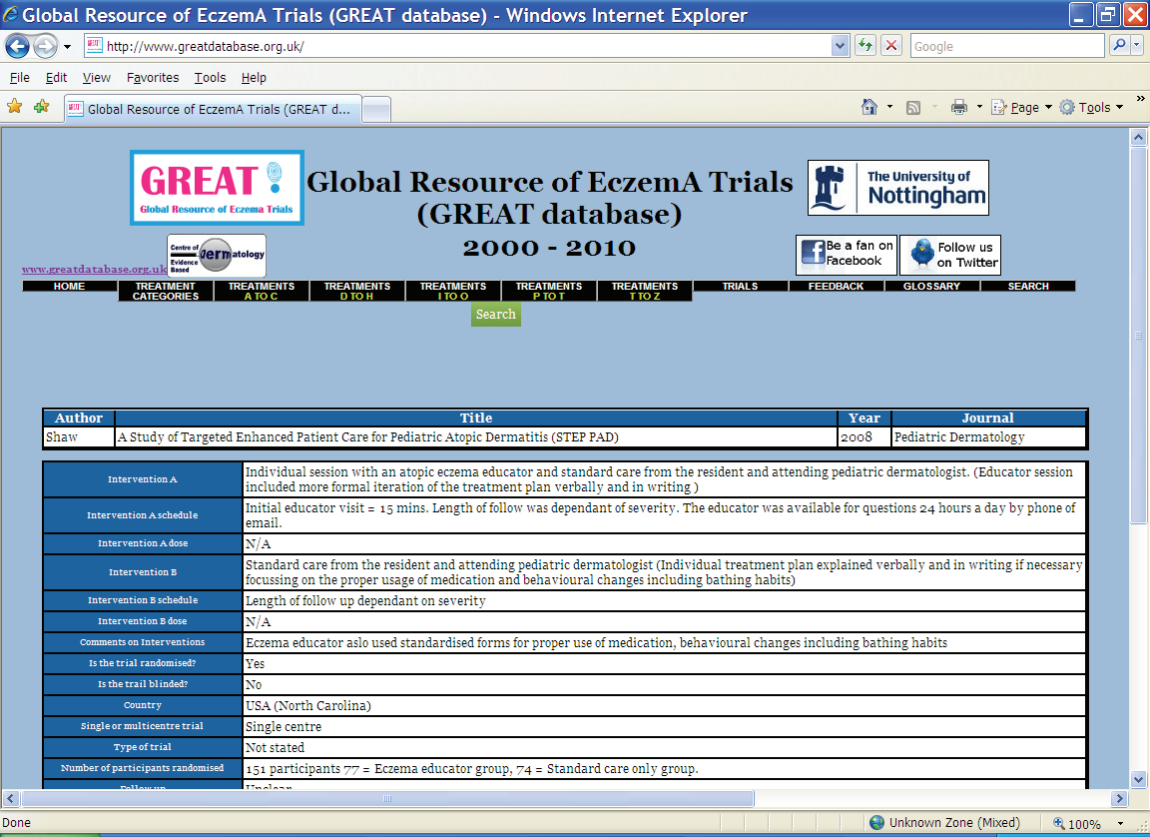
**The GREAT database - Individual trial information**.

For each trial, the database gives the citation for the published report. As well as this it provides information on the trial in a clear and simple format. There is basic information about the interventions, study design and methodology (such as use of blinding and intention-to-treat methods), number of participants randomized, withdrawals, outcomes, authors' conclusions, sponsorship, conflicts of interest and quality of reporting (Figure 3).

A further, deeper level of data on each trial in the GREAT database has been extracted including the results of efficacy outcomes with all associated numerical data. This opens up the possibility of our team collaborating with interested groups who need access to properly extracted data on eczema treatments to speed up the production of research such as systematic reviews and clinical guidelines. Interested groups should contact the authors to discuss this in more detail.

The information provided aims to allow users of the GREAT database to quickly and easily decide if the trial is of interest to them. The complete citation(s) for each trial are given to facilitate obtaining the full paper for further scrutiny.

### Cochrane Central Register of Controlled Trials (Clinical Trials)

The only other database which contains records of RCTs of eczema (as well as RCTs on all other medical subjects) and is updated regularly is Clinical Trials. The Clinical Trials database gives details of RCTs and controlled clinical trials (CCTs) on eczema treatment taken from MEDLINE, EMBASE and other databases along with the Cochrane Groups' specialised registers, which include citations found by handsearching. The Cochrane Skin Group Specialised Register has been collecting full paper copies of CCTs and RCTs for 12 years.

The information given on each trial in Clinical Trials often includes the abstract and may allow the user to retrieve the full paper. The Clinical Trials database gives a very thorough list of published and unpublished reports of trials and is useful for those interested in CCTs as well as RCTs, but it does not give any extracted information. By comparison, the GREAT database gives enough information to usually save having to retrieve the full paper for every single eczema trial. Also, if only interested in eczema, skills in database searching would be required to be sure to obtain a specific and fully comprehensive list of only eczema trials from the Clinical Trials database.

The GREAT database provides eczema researchers with a very specific tool which can be used alongside resources such as the Clinical Trials database.

### The future of the GREAT database

For those in need of instant access to a list of RCTs published between 2000 and 2010 on eczema treatments the GREAT database now meets this need, however, RCTs before and after this time period may still require some hours of perseverance to retrieve citations and papers of interest.

For pre-2000 trials, help is at hand from the HTA systematic review on treatments for eczema [12]. This provides full citations with some relevant and useful information. There is, however, still significant effort involved in wading through this document for each trial, with no facility to quickly search for your area or trial of interest. One way to extend the usefulness of this resource in the future will be to provide information for RCTs before 2000 though the GREAT database.

The search used to identify the RCTs included in the database currently runs to the end of 2010, however, the GREAT database would quickly lose some of its usefulness if there were no subsequent searches added. One of the authors of the database (HN) will be undertaking regular update searches until the end of 2013, when the current project ends. It is the aspiration of the UK Centre of Evidence-Based Dermatology to continue the work of keeping the information in the database up to date past this point and well into the future and to encourage and facilitate other research groups around the world to prepare and maintain similar international trial collections for other important skin diseases such as psoriasis, melanoma and vitiligo.

Evaluation of the database against the systematic reviews included in the 2009 and 2010 annual evidence updates produced by NHS evidence - skin disorders [7] found that all but a few of the RCTs listed in the citations were included in the GREAT database. Many RCTs included in the GREAT database were not found in either the included or excluded studies of the relevant systematic reviews.

An advantage of the GREAT database is that it can and will be updated, so all RCTs found can be quickly added. To ensure that missed RCTs are found, users of the database are encouraged to alert the authors to RCTs not currently included in GREAT.

## Conclusions

The Global Resource of Eczema Trials has been created to speed up eczema research. The collective time saved by research groups around the world can now be used to make strides in optimising the treatment of eczema, in order to further benefit people with eczema. This up to date resource fills a gap which no other resource covers in such detail. The database will also result in health gains as knowledge locked in obscure RCTs is liberated freely to health care professionals working in clinic, or indeed to patients who wish to investigate the evidence for a particular treatment. The database will continue to be expanded in order to secure its place as the number-one resource of randomized controlled trials on eczema treatment both now and in the future.

## Availability and requirements

The database is freely available online at http://www.greatdatabase.org.uk

## Competing interests

HN is currently studying for a PhD which is being supported by the grant for this project. AM has no competing interests. HCW has no competing interests.

## Authors' contributions

HN filtered the search and obtained the papers, with assistance from HCW. HN extracted the data from each trial and entered this into the database. HCW conceived and developed the idea of the database and the name. HCW provided expert comments and guidance on eczema, and eczema research. HN conducted the evaluation of the database with assistance from AM. AM provided an analysis of the database. All authors read and approved the final version of the paper.

## Pre-publication history

The pre-publication history for this paper can be accessed here:

http://www.biomedcentral.com/1471-5945/11/10/prepub
